# Complementary Medicine, Exercise, Meditation, Diet, and Lifestyle Modification for Anxiety Disorders: A Review of Current Evidence

**DOI:** 10.1155/2012/809653

**Published:** 2012-08-27

**Authors:** J. Sarris, S. Moylan, D. A. Camfield, M. P. Pase, D. Mischoulon, M. Berk, F. N. Jacka, I. Schweitzer

**Affiliations:** ^1^Department of Psychiatry, The University of Melbourne, Melbourne, VIC 3000, Australia; ^2^Centre for Human Psychopharmacology, Swinburne University of Technology, Melbourne, VIC 3122, Australia; ^3^NICM Collaborative Centre for Neurocognition, Melbourne, VIC 3122, Australia; ^4^Deakin University, Geelong, VIC 3220, Australia; ^5^Depression Clinical and Research Program, Department of Psychiatry, Massachusetts General Hospital, Harvard Medical School, Boston, MA 02115, USA; ^6^Mental Health Research Institute, Parkville, VIC 3052, Australia; ^7^Orygen Youth Health Research Center, Parkville, VIC 3052, Australia

## Abstract

Use of complementary medicines and therapies (CAM) and modification of lifestyle factors such as physical activity, exercise, and diet are being increasingly considered as potential therapeutic options for anxiety disorders. The objective of this metareview was to examine evidence across a broad range of CAM and lifestyle interventions in the treatment of anxiety disorders. In early 2012 we conducted a literature search of PubMed, Scopus, CINAHL, Web of Science, PsycInfo, and the Cochrane Library, for key studies, systematic reviews, and metaanalyses in the area. Our paper found that in respect to treatment of generalized anxiety or specific disorders, CAM evidence revealed current support for the herbal medicine Kava. One isolated study shows benefit for naturopathic medicine, whereas acupuncture, yoga, and Tai chi have tentative supportive evidence, which is hampered by overall poor methodology. The breadth of evidence does not support homeopathy for treating anxiety. Strong support exists for lifestyle modifications including adoption of moderate exercise and mindfulness meditation, whereas dietary improvement, avoidance of caffeine, alcohol, and nicotine offer encouraging preliminary data. In conclusion, certain lifestyle modifications and some CAMs may provide a beneficial role in the treatment of anxiety disorders.

## 1. Introduction

Anxiety disorders as a collective entity are pervasive and include discrete diagnoses of generalized anxiety disorder (GAD), social phobia (SP), obsessive compulsive disorder (OCD), panic disorder (PD), and post traumatic stress disorder (PTSD) [1]. Anxiety disorders present with a marked element of psychological tension and distress and are accompanied by a range of somatic symptoms such as palpitations, shortness of breath, dizziness, hyperthermia, and digestive disturbance [1]. Lifetime prevalence rates of anxiety disorders are approximately 3%–6% for GAD, 4%–6% for SP, 1%–3% for OCD, 1%-2% for PTSD, and 1%–3% for PD [2, 3].

 There are currently various effective psychological therapies and pharmacological treatments available for anxiety disorders. The recommended first-line treatment strategies for most anxiety disorders include antidepressants and/or cognitive behavioural therapy (CBT) [4]. In respect to pharmacotherapies, there is a good evidence base for both short-term and long-term treatment with paroxetine (especially for PD), escitalopram, venlafaxine extended release, and duloxetine [5]. Despite their modest effectiveness (effect size for GAD of 0.38 for selective serotonin reuptake inhibitors: SSRIs) [6], antidepressants have significant limitations, including a relatively slow onset of action and time to maximal effect, and a number of possible side effects, including initial increase in anxiety in the short-term (often problematic for patient compliance), and sexual side effects, which affect over 50% of users [7] in the longerterm. 

 Benzodiazepines have established efficacy for many anxiety disorders [5] but also numerous limitations. They can be very helpful for ameliorating symptoms rapidly and are usually prescribed for short-term use. However, this recommendation can be very difficult to institute due to potential reliance for symptom relief. Benzodiazepines are no longer recommended beyond short-term use due to potential for abuse, the development of dependence, significant risks when combined with alcohol, and possible long-term cognitive effects [8]. There have been recent positive randomised controlled trials (RCTs) with pregabalin, however, its indication for GAD in Europe has not yet been replicated by other regulatory authorities [5]. Psychological techniques are also effective first-line interventions for anxiety [9]. In particular, there is a body of evidence for CBT that usually involves psychoeducation, relaxation training, cognitive restructuring and behavioural aspects [4]. Despite effectiveness, some patients are not suited or motivated for face-to-face CBT, access may be an issue, and the cost can be prohibitive [10].

 Approximately 50%–65% of patients with anxiety disorders benefit from CBT or antidepressants [11, 12]. However, many patients continue to suffer significant symptoms despite treatment, underlining the need for further options or adjuncts to current conventional treatments. Furthermore, anxiety disorders are often under-treated [13], motivating patients to seek different treatment approaches such as complementary and alternative medicine (CAM). Non-conventional treatments are commonly used for anxiety, with CAM, lifestyle modifications, and self-help techniques often used in concert with pharmacotherapies and psychological techniques [14, 15]. Complementary medicines consist of herbal and nutrient products, while complementary therapies include interventions such as acupuncture, naturopathy, chiropractics, and homeopathy [16]. Self-help techniques include Tai chi, yoga, and meditation, while lifestyle modifications may involve the employment of dietary alteration, exercise, and the minimisation of substances such as alcohol, caffeine, and tobacco. CAM use for people with anxiety disorders is prevalent with a US cross-sectional and longitudinal survey demonstrating 43% of individuals with DSM-IV criteria for GAD, PD, SP, or PTSD use a variety of CAM treatments [17]. Use of CAM was associated with a diagnosis of GAD, older age, greater education, and having two or more chronic medical conditions.

While previous reviews have explored the use of CAM or self-help techniques in the management of anxiety [14, 18, 19], to date no review has comprehensively assessed this broad area across all major clinical anxiety disorders to provide an integrated assessment of current evidence. Thus, this narrative paper examines the current evidence base for nonconventional treatments of anxiety disorders, including discussion of their neurobiological underpinnings, and provides considerations for their potential integration into clinical practice.

## 2. Methods

A range of databases (PubMed, Scopus, CINAHL, Web of Science and PsycINFO, and The Cochrane Library) were searched during early 2012. Each author contributed to an area of their respective expertise (D. Mischoulon: CAM therapies; J. Sarris and M. P. Pase: CAM natural products (i.e., herbal and nutrient supplements); F. N. Jacka and S. Moylan: diet and exercise; D. A. Camfield: meditative techniques; M. Berk lifestyle modification; I. Schweitzer: conventional pharmacotherapies). The literature search of key interventions for treating general anxiety, and diagnosed clinical anxiety disorders (GAD, PD, SP, OCD, and PTSD) focused on (1) CAM natural products (nutraceuticals) (2) CAM modalities (3) Meditation and mindfulness techniques (4) Lifestyle modification using physical activity, exercise, diet, and restriction of caffeine, alcohol, and nicotine (see Table 1). As a narrative inclusive “metareview” was conducted, the studies included a range of evidence (from epidemiological to human clinical studies, and metaanalyses and systematic reviews). Key studies, reviews, and metaanalyses were focused on. Further review of the literature was undertaken to study the posited mechanisms of action of these interventions. The literature reviewed was restricted to papers written in English. The term “significant” was applied to results with a *P *value of <0.05.

## 3. Results

### 3.1. Complementary Therapies

#### 3.1.1. Overview

There are several complementary therapy modalities touted as having potential benefit in the treatment of anxiety, including interventions such as acupuncture, massage, naturopathy, chiropractics, and homeopathy [16]. Regulation of these practices and their practitioners vary depending on jurisdiction, and if clinicians are referring patients for these treatments, several considerations should be recognised, including evidence for the modality, cost of the treatment, and the training and qualification of the therapist. 

#### 3.1.2. Naturopathic Medicine

Naturopathic practitioners seek to improve health by assisting the body's innate capacity to recover from illness [20]. Practitioners typically employ a combination of many CAM therapies, including herbal and nutritional supplementation, and dietary and lifestyle recommendations, and may additionally incorporate acupuncture or homeopathy. Only one study has been performed with regard to naturopathic medicine (naturopathy) in the area of anxiety disorders, although another naturalistic observational study is currently underway [21]. 

A double-blind RCT by Cooley and colleagues [22] compared a naturopathic therapy (NT) protocol, including the herbal medicine Ashwagandha (*Withania somnifera*) 300 mg twice daily, dietary counseling, breathing relaxation techniques, and a standard multivitamin. Eighty-seven subjects with moderate to severe anxiety of longer than six weeks were recruited. The control group received a standardized psychotherapy intervention (PT), matched deep breathing relaxation techniques, and placebo pill supplement over 12 weeks. Final Beck Anxiety Inventory (BAI) scores decreased significantly in both groups, by 56.5% in the NT group and 30.5% in the PT group, with a highly significant advantage for the NT group compared to PT group. The NT group also had significantly greater clinical benefit in mental health, concentration, fatigue, social functioning, vitality, and overall quality of life. No serious adverse reactions were reported in either group. The study, while enlightening, was limited by a small sample size, and utilisation of comprehensive treatment packages, making it difficult to assess the benefit of any particular component. Additionally, Naturopaths frequently apply complex personalized herbal and nutritional formulations in treatment protocols, and thus the standardized intervention does not reflect normal practice. The results however are encouraging and endorse further research.

#### 3.1.3. Massage Therapy

Therapeutic massage is one of the most popular CAM treatments for anxiety, but has had few rigorous evaluations for diagnosed anxiety disorders. A recent study by Sherman and colleagues [23] assessed the effectiveness of massage for treatment of GAD. The investigators randomised 68 patients with GAD to therapeutic massage, thermotherapy (the application of heat to the body), or relaxation-room therapy for 12 weeks (total 10 sessions). Changes in various outcome measures, such as the Hamilton Anxiety Rating Scale (HAM-A), were assessed at the conclusion of the 12 weeks and also after six months of additional followup. All three treatment groups had significant improvement on the HAM-A after 12 weeks and maintained their gains after 26 weeks. However, no significant differences were found between groups. The authors concluded that all three treatment arms showed some clinically significant improvements, perhaps due to a generalized relaxation response rather than any individual characteristics of the three treatments. In fact, given the lower expense of the relaxation-room, the authors suggested that this particular intervention might prove the most cost-effective for GAD patients who are interested in CAM [23].

 Other investigations have compared CBT against aromatherapy massage (AM) for treating symptoms of anxiety or depression in cancer patients. In a sole comparison of these two interventions, Serfaty and colleagues [24] recruited 39 cancer patients with anxiety and/or depression assessed on the Hospital Anxiety Depression Scale (HADS). Subjects were randomised to treatment as usual (TAU) plus up to eight sessions of AM or CBT over three months. AM proved the more acceptable therapy based on sessions attended, and both treatments were significantly effective based on the Profile of Mood States (in respect to total mood, depression, and anxiety scores). CBT had a trend to an advantage in depression but not in anxiety.

Black and colleagues [25] performed the first controlled study of chair massage for anxiety in 82 inpatients withdrawing from alcohol, cocaine, and opiates. Patients were randomised to three days of chair massage or a relaxation control condition. Standard counseling and pharmacologic management were offered concurrently to all patients. Spielberger State-Trait Anxiety Inventory (STAI) scores were reduced for both interventions, with a significant advantage for chair massage.

In summary, preliminary results for massage therapy suggest benefit in various different patient populations and contexts, but results are not conclusive, nor commonly employ a clinical population, while comparisons with control interventions have often not suggested significant differential effects. Additional research will be necessary to characterize their place in anxiety disorder treatment.

#### 3.1.4. Acupuncture

Acupuncture is a very popular CAM intervention that, unlike the previously mentioned interventions, has a more substantive body of clinical trial evidence regarding its efficacy and safety in the treatment of anxiety and depression. A recent review by Pilkington et al. [26] identified 10 randomized and two nonrandomised clinical trials of acupuncture for generalised anxiety or anxiety disorders. Among the RCTs, four were in patients with GAD or anxiety neurosis, and six in patients with acute perioperative anxiety.

Analysis of the studies was hampered by common problems encountered when interpreting the acupuncture literature in general. These include between-trial variation in acupuncture protocols and specific points used. Furthermore, different studies used a variety of control interventions, including behavioral desensitisation, sham acupuncture with nonspecific points, biofeedback, and drug therapy. The authors were rightfully suspicious of some of the reported “cure rates” that they described as “unrealistically high”. Acupuncture appeared to be comparable in efficacy to drug therapy, but this similarity could be explained by the small sample sizes leading to underpowering of studies, limiting the ability to detect a difference between treatments. Other limitations included the mixing of diagnoses in the samples, for example, anxiety disorder and depression. 

Regarding studies for specific DSM-IV diagnoses, Pilkington and colleagues [26] identified 3 trials specifically on GAD. These trials were all of short duration (four to six weeks) and compared acupuncture against pharmacotherapy. Similar efficacy was found for both interventions, but small sample sizes limited the generalisability of the findings. Very few trials in other anxiety disorders such as OCD and PTSD have also been published, but due to design flaws do not support solid recommendations about potential efficacy. In summary, acupuncture appears effective in short-term use for anxiety symptoms, but we cannot yet make clear recommendations for specific clinical anxiety disorders. Adjunctive therapy studies are lacking, which is unfortunate given that acupuncture is generally well tolerated and does not appear to cause adverse interactions with pharmacological therapies [27]. 

Acupuncture has been documented to interact with opioid pathways, and interventions which modify these pathways have been shown to have potential mood modulatory activity [28, 29]. Other potential mechanisms of action that may induce anxiolysis include increased release of serotonin and norepinephrine, and cortisol modulation [30]. Examination of biological mechanisms of acupuncture and massage therapy, including measurements of neurotransmitters and immune biomarkers has been conducted in the area of depression [26, 31], although specific research into the underpinning neurobiological effects of these interventions to treat anxiety has not yet been explored. 

#### 3.1.5. Homeopathy

The practice of homeopathy (use of preparations consisting of highly diluted substances) has its roots in the 1800s in Germany where it was used to treat a range of illnesses and health disorders [16]. A systematic review by Pilkington and colleagues [32] identified eight RCTs using homeopathy to treat anxiety. Results revealed that the benefit of homeopathy in generalised anxiety and anxiety disorders is unable to be determined, as the identified studies all exhibited significant methodological issues (e.g., small samples, lack of control group). Reported adverse effects appear to be limited to “remedy reactions” and included temporary worsening or reappearance of minor symptoms. Another stricter systematic review by Davidson et al. [33] of homeopathy in psychiatry, found that for four of five studies for anxiety (two on GAD), homeopathy was not superior to placebo. An example of an RCT using homeopathy to treat anxiety was conducted by Bonne and colleagues [34]. Forty-four patients with diagnosed GAD participated in a 10-week RCT of individually tailored homeopathic remedy. While significant improvement in most measures, including HAM-A, was observed in both the active treatment and placebo groups, no difference was found between groups. In summary, on the basis of the current literature it is not possible to provide support for the use of homeopathy for treatment of anxiety disorders. 

#### 3.1.6. Conclusions

At this time there is preliminary evidence for CAM therapies including acupuncture, massage, and naturopathy in the treatment of anxiety disorders; while homeopathy is not supported by clinical evidence. No studies identified assessed chiropractics or osteopathy in treating anxiety disorders. Thus, in the absence of conclusive studies, clinicians should prescribe these treatments with caution as their efficacy is not as well characterised as more standard therapies. 

### 3.2. Nutraceuticals (Herbal and Nutritional Medicine)****

#### 3.2.1. Overview

Over the past 25 years, there has been increasing interest in the use of nutraceuticals (herbal or nutritional medicines) for the treatment of anxiety disorders, with numerous human clinical trials emerging in the area [19, 35, 36]. At present, the clinical evidence for herbal medicines, nutrients, and aromatherapy in the treatment of anxiety varies greatly in respect to methodological quality (see Table 2). Considerations when referring or prescribing nutraceuticals involve the knowledge of potential drug interactions (e.g., with *Hypericum perforatum* (St John's wort) [37]), the additional cost of these supplements, and most importantly, concern over quality issues [38]. While some nutraceutical companies provide researched, standardised products, manufactured to a pharmaceutical standard, such confidence cannot be extended to many products, thus clinicians need to be mindful that quality and safety are paramount when integrating nutraceuticals into the therapeutic domain. 

#### 3.2.2. Clinical Evidence for Herbal Medicines

The most researched herbal medicine in the treatment of anxiety is* Piper methysticum* (Kava); a perennial plant native to various regions of the South Pacific [62]. The roots of the Kava plant are traditionally prepared as a water-based beverage for its medicinal properties and psychotropic effects [63]. In 2003, a Cochrane review evaluated the effectiveness of Kava in treating anxiety [42]. Out of 12 RCTs that satisfied the inclusion criteria, seven used the HAM-A and were included in the metaanalysis. The pooled analysis found Kava to be effective in reducing the HAM-A total score relative to placebo (weighted mean difference = 3.9, *n *= 380). Another pooled analysis of six studies found an effect size of 1.1 (Cohen's *d*) in favour of Kava reducing HAM-A score over placebo [64]. Those studies not included in the pooled analysis were generally congruent with this result. A further metaanalysis of six RCTs reported that a standardised Kava extract (WS1490) was effective in significantly improving anxiety relative to placebo (by 5.94 points on the HAM-A) further supporting the anxiolytic effects of Kava [43]. The anxiolytic properties of Kava appear to be due to a collective group of compounds called kavalactones [65]. Amongst numerous mechanisms, kavalactones appear to modulate calcium and sodium channels [66], modify binding of ligands to GABA receptors [67] and inhibit noradrenaline uptake [68]. 

 Despite evidence of efficacy, support for the use of Kava has been blunted by ongoing safety concerns following numerous reports of liver toxicity [69]. As discussed in detail elsewhere [64], various factors such as the manufacturing quality, plant part of Kava used, the method of extraction, and the dose are potential in factors that affect the safety of Kava [70]. Interactions with alcohol and pharmaceutical medications should also be considered.

Limited evidence suggests that *Bacopa monnieri *(Bacopa) [71] and *Ginkgo biloba* (Ginkgo) [35], both used for their cognitive enhancing effects, may have anxiolytic properties in humans. Although Ayurvedic writings document Bacopa in the treatment of anxiety [72], double-blind RCTs investigating effects of Bacopa on anxiety are scarce. In one study, where cognition was the primary outcome, 300 mg of standardised Bacopa extract daily for three months in 46 healthy volunteers was found to reduce State anxiety relative to placebo [39]. One double-blind RCT investigated the effects of Ginkgo extract EGb 761 on anxiety [41], using the HAM-A as the primary outcome measure in 107 patients with GAD or adjustment disorder with anxious mood. After four weeks of supplementation both a high (480 mg/day) and low dose (240 mg/day) of EGb 761 significantly reduced anxiety compared to placebo in a dose-dependent fashion.

 Commonly considered an anti-depressant, the anxiolytic effects of St John's wort were investigated in a double-blind multicentre RCT using a cohort of 324 outpatients with mild to moderate depression. Twice daily supplementation with 250 mg of St John's wort, relative to 75 mg of imipramine, significantly improved scores on the HAM-A somatisation subscale [73] over a period of six weeks. A further 6-week multicentre double-blinded RCT reported that St John's wort (600 mg/day) significantly improved various subscales on the HAM-A in a cohort of 151 outpatients with somatoform disorders [51]. However, studies exploring the use of St John's wort in specific anxiety disorders have not produced positive results. For example, two smaller double-blind RCTs have not supported its use (both trials used a flexible dose of 600–1800 mg extract daily over 12 weeks) in reducing the symptoms of OCD [49] or SP [50]. Due to this, St John's wort cannot be recommended as an anxiolytic for use in any anxiety disorder.

 Limited evidence exists for an anxiolytic effect of *Scutellaria lateriflora* (Scullcap) and *Passiflora incarnata* (Passion flower), both of which have traditionally been used in the Americas as calming agents [74]. Following acute administration, one small study reported that Scullcap reduced anxiety relative to placebo in a dose-dependent fashion [48], however, the methodology was weak and the results were communicated poorly. Building upon promising preclinical research [75], Akhondzedah et al. [47] reported that 45 drops of randomised Passion flower extract was comparable to oxazepam in reducing anxiety in 36 patients with GAD. Two acute double-blind RCTs (samples of 60 participants) have reported that 500 mg of the herbal medicine significantly reduced anxiety associated with surgery relative to placebo [46, 76]. Importantly, no negative interactions with anesthesia and surgical outcomes were observed.

 A single double-blind RCT has assessed the use of* Matricaria recutita* (Chamomile) in the treatment of GAD [40]. Following eight weeks of treatment in 57 patients, Chamomile was found to have a modest effect on reducing anxiety (HAM-A scores) relative to placebo. Adverse events were not different between the placebo and treatment groups. Several small double-blind RCTs suggest that *Melissa officinalis *(Lemon balm) increases self-rated calmness acutely in healthy participants [44, 45]. Interestingly, all three of these double-blind studies reported significant effects on self-rated calmness with different dosages of Lemon balm (ranging from 300–1600 mg). The effects of Lemon balm have yet to be investigated in clinical samples. 

 Aromatherapy (the practice of prescribing isolated volatile oils from plants via inhalation, massage, or internal use) is one of the most widely used CAMs in the treatment of anxiety. A recent systematic review concluded that, across the 16 studies, aromatherapy was generally safe and effective in reducing anxiety symptoms [61]. The review reported that those with higher levels of anxiety appeared to respond better to aromatherapy than those with mild anxiety. However, the review noted that many of the studies were of questionable quality and study cohorts were heterogeneous, comprising participants with dementia, cancer, and also healthy volunteers. 

#### 3.2.3. Clinical Evidence of Nutrients

 At present, there is little evidence to support the anxiolytic effects of nutrient supplementation for treatment of most anxiety disorders (see Table 2). One large double-blind RCT (*n *= 264) reported that supplementation with magnesium, when combined in a herbal formula, decreased anxiety in patients with mild to moderate generalised anxiety over three months relative to placebo [77]. In relatively healthy samples, multinutrient therapy has been shown to have no effect on state anxiety (*n *= 205) over 12 months [57]or self rated calmness following 28 days (*n *= 198) [56] and 33 days (*n *= 215) [55] of treatment. However, while approximately one month of multinutrient supplementation reduced anxiety relative to placebo across 88 healthy males [52] and 44 women with premenstrual symptoms [53], a further study reported no beneficial effect of multinutrient therapy on anxiety in participants in aged care following eight weeks of supplementation [54]. In healthy participants (*n *= 108), one double-blind RCT reported that a week of amino acid treatment (2.64 g L-lysine and 2.64 g L-arginine per day) decreased stress-related anxiety [78].

 Despite a lack of evidence for nutrient supplementation in other anxiety disorders, evidence does suggest that inositol may have a potential role in ameliorating panic. In 21 patients with diagnosed PD with or without agoraphobia, 12 g/day of inositol was shown to quell the frequency and severity of panic attacks and the severity of agoraphobia in a double-blind four week cross-over trial [58]. A later small trial involving 20 participants reported that Inositol (18 g/day for one month) was more effective than fluvoxamine (250 mg/day for one month) in reducing weekly panic attacks [60]. In 13 patients with OCD, 18 g/day of Inositol reduced OCD symptoms in a six week cross-over trial [59], however other studies have revealed equivocal effects to placebo [79]. 

#### 3.2.4. Mechanisms of Action

The proposed pharmacodynamics of herbal medicines used for treatment of anxiety disorders primarily involve modulation of neuronal communication, affecting neuroreceptor binding and activity [35], or alteration of neurotransmitter synthesis and activity [36]. Anxiolytic nutraceuticals may have effects on the GABA system either via inducing ionic channel transmission by voltage-gated blockage, or through alteration of membrane structures, GABA transaminase or glutamic acid decarboxylase inhibition, or less commonly via binding with benzodiazepine receptor sites (e.g., GABA-A) [35]. Other actions may involve dampening CNS activity, and regulating or supporting the healthy function of the hypothalamic pituitary adrenal-axis [80].

#### 3.2.5. Conclusions

In summary, for reducing generalised anxiety, evidence supports the use of Kava (although potential extremely rare hepatotoxicity needs to be considered), while limited research points towards a beneficial effect of Ginkgo, Passion flower, and Chamomile, multinutrient formulations, Scullcap, Lemon balm, and Bacopa. Further studies are needed to extend this research into clinical cohorts. Although there appears to be growing support for a general anxiolytic effect of aromatherapy, future research is required to explore the effects on clinical anxiety disorders. There is little support for the use of St John's wort for any anxiety disorder, while tentative evidence supports inositol for PD and discourages its use in OCD.

### 3.3. Physical Activity and Exercise

#### 3.3.1. Overview

Physical inactivity is an established risk factor for the development of many of health disorders, including psychiatric illnesses [81]. For example, increased rates of MDD were correlated with physical inactivity in surveys of German [82] and Norwegian populations [83]. Regular physical activity appears to reduce all-cause mortality and is an established independent protective factor for premature death [84, 85]. Mortality and morbidity aside, regular physical activity has been repeatedly shown to be associated with improved emotional wellbeing [86] and inactivity with poorer emotional wellbeing [87]. 

#### 3.3.2. Epidemiological Evidence

There is now considerable evidence drawn from epidemiological surveys demonstrating the association of regular physical activity with reduced depressive and anxious symptoms [88, 89]. Utilising cross-sectional data from the US national comorbidity study of 8098 adults aged 15 to 54, Goodwin [90] revealed regular physical activity as being associated with decreased prevalence of current anxiety disorders, including panic attacks (OR: 0.73), SP (OR: 0.63), specific phobia (OR: 0.78), and agoraphobia (OR: 0.64). However, an inherent issue with these cross-sectional studies is their inability to explain the direction of relationship between physical activity and anxiety. Regardless, mental disorders characterised by anxiety and depression are associated with many symptoms which may lead to reduced physical activity, such as fatigue, poor motivation and social isolation [91].

#### 3.3.3. Clinical Evidence

Few prospective observational studies have been conducted assessing the impact of physical activity on anxiety disorders. Ströhle et al. [92] demonstrated a lower incidence of some anxiety disorders, and all anxiety disorders when grouped (OR: 0.70), as being associated with regular physical activity in a sample of 2548 adolescents and young adults. In a Swedish cohort prospectively followed for 2 years, moderate to vigorous physical activity (defined as >2 hours of activities per week, including aerobics, dancing, swimming, playing football, or gardening) was significantly associated with fewer symptoms of anxiety on the HADS (adjusted RR: 0.56) [93]. In this study, the association between physical activity and depression was of greater magnitude than with anxiety; a finding consistent with previous studies in which self reported depression rates (but not anxiety) were associated with less physical activity [94, 95]. Prescriptive exercise has demonstrated efficacy as a treatment strategy for anxiety symptoms. In a recent metaanalysis, exercise as a therapeutic intervention was found to be as effective as psychotherapy, and nearly as effective as pharmacotherapy, for the treatment of self-reported anxiety symptoms [96]. The effect size of exercise for reduction of anxiety symptoms (*d* = −0.48) however appears smaller than the benefit observed in RCTs of exercise therapy in clinical depression (*d* = 1.42) [97].

In regards to prescriptive use of exercise in diagnosed anxiety disorders, currently there is a surprising deficit of robust studies. However, in PD, a study of 46 individuals suffering from moderate to severe PD reported that patients who received structured aerobic exercise had significantly greater symptomatic improvement than patients who received placebo, but less improvement than those receiving clomipramine [98]. Further, aerobic exercise training was not statistically superior to relaxation when combined with either paroxetine or placebo in the treatment of patients with PD [99]. Other available pilot studies of structured exercise programs have demonstrated beneficial effects as individual or adjunctive therapies in patients with PTSD [100–102], SP [103] and OCD [104]. However, these studies generally suffer from methodological issues including lack of control groups and small sample sizes. As it stands, little other RCT evidence exists supporting the efficacy of exercise in the treatment of clinical anxiety disorders, although further evidence may soon become available [105, 106].

#### 3.3.4. Mechanisms of Action

The therapeutic effect of physical activity or exercise in reducing anxiety is likely the result of complex interactions between developmental, neurobiological, and psychological processes [107]. The neurobiological mechanisms underpinning the effect of physical activity on anxiety are not yet fully elucidated, but potentially involve modulating activity of 5-HT neurons in the dorsal ralph nucleus (DRN), modulation of the autonomic nervous system (ANS) and regulation of various neuroactive agents, including brain derived neurotrophic factor [108] and beta-endorphins [109], and atrial natriuretic peptide [110]. Exercise appears to protect against stress-induced sensitisation of 5-HT DRN neurons which are known to exhibit afferent and efferent projections to cortical regions, the amygdala, locus coeruleus (LC), and periaqueductal grey matter, which are areas associated with fear, anxiety, and mood regulation [111]. In addition, exercise is known to produce an anti-inflammatory state [112] and potentially increase production of brain antioxidants [113]. Inflammation and oxidative stress are both postulated to have roles in the pathogenesis of numerous mental disorders including major depression [114]. In PD, exercise may be beneficial by inducing interoceptive cues that elicit anxiety, and by this repeated exposure to those sensations may assist in training the person to manage these symptoms [115]. Furthermore, exercise may induce beneficial downregulation of 5-HT2C receptors in sufferers of PD [116, 117]. It is possible these factors interact in a complex fashion in exerting protective effects on the tumescence of anxiety symptoms.

#### 3.3.5. Clinical Recommendations

Current clinical practice guidelines for the treatment of anxiety disorders including, PD, [118] support regular physical activity (e.g., walking for 60 minutes or running 20–30 minutes at least four days per week) as part of best-practice treatment. These recommendations are the same as those for MDD [119]. This is prudent given the high comorbidity between depression and anxiety. Moreover, given the relationship between physical activity and mental health appears to be complex [120], the input of robust RCT evidence will assist in substantiating and refining these recommendations. For example, some evidence suggests exercise recommendations should differ between men and women. In a survey assessing the relationship between exercise intensity and mental health, the positive effects of vigorous activity on anxiety symptoms found in men were not replicated in women [120]. In contrast to men, women appeared to benefit significantly from less intense forms of exercise (e.g., walking), with benefits found in more general domains (e.g., well-being and somatisation) and not those directly related to depressive or anxiety symptoms.

#### 3.3.6. Conclusions

The weight of evidence supports the use of moderate graded exercise to reduce anxiety and can be in most cases recommended after an appropriate health assessment. While the evidence strongly supports PA and exercise as an acute anxiolytic, the utility of exercise as treatment for anxiety disorders would benefit from rigorously controlled studies.

### 3.4. Meditation Techniques (Mindfulness, Yoga, Tai Chi) 

#### 3.4.1. Overview

The concept of meditation is varied, with a key attribute of the practice involving “mindfulness,” commonly defined as the awareness which arises through “paying attention in a particular way: on purpose, in the present moment, and nonjudgmentally” [121]. The development of mindfulness has its origins in the spiritual tradition of Buddhism, which dates back over 2,500 years. Over the past 60 years, ever since the advent of Buddhism in the West, there has been growing interest in the relationship between mindfulness, meditation, and mental health. In regards to anxiety disorders, a growing body of empirical evidence exists. The use of meditation as a Western behavioral intervention was pioneered by Kabat-Zinn [122], who originally investigated structured mindfulness training for the treatment of chronic pain. The training program has become known as Mindfulness-Based Stress Reduction (MBSR). MBSR is an 8–10 week structured program which involves (i) training in mindfulness meditation practice, (ii) mindful awareness, for example, during yoga postures, and (iii) mindfulness during stressful everyday situations and social interaction. The typical MBSR course involves weekly group meetings, a one-day workshop, and daily individual practice at home [123]. Over the past 30 years, the MBSR technique has been investigated in relation to a number of physical and mental illnesses, with a moderate clinical effect being reported in relation to the effect of MBSR on general mental health [123]. There is also evidence to suggest that amongst the general population, the MBSR technique may bring about a reduction in trait and state anxiety and symptoms [124, 125]. A number of mindfulness techniques in the context of adaptations of CBT have also been developed, including Mindfulness-Based Cognitive Therapy (MBCT) [126], Dialectical Behavior Therapy (DBT) [127], and Acceptance and Commitment Therapy (ACT) [128]. However, due to the vast quanta of data in the area, the focus of the current section will be on formal mindfulness meditation techniques rather than obscure techniques, or specific mindfulness approaches to CBT.

#### 3.4.2. Clinical Evidence

Kabat-Zinn et al. [129] first investigated the efficacy of an 8-week MBSR program on anxiety scores in 22 patients who met DSM-IIIR diagnostic criteria for GAD or PD, as well as 58 “non-study” participants who did not meet formal criteria for an anxiety disorder yet scored above the 70th percentile on the Symptom Check List-90 Revised (SCL-90-R). At the end of the treatment period, as well as at 3-month followup, the number of patients experiencing panic symptoms (measured by the Hamilton Panic Score), was found to be significantly reduced, together with overall symptoms of anxiety. In a followup study, 18 of the original patients in addition to 39 of the larger study comparison cohort we assessed by Miller [130] in order to determine if the improvements in anxiety symptoms were maintained at 3 years. In the 18 original patients, posttreatment improvements in the Hamilton Panic Score, as well as the number and severity of panic attacks were maintained at 3-year followup, together with improvements on anxiety outcome measures. 

As an adjunct to pharmacotherapy with benzodiazepines or SSRIs, Lee et al. [131] investigated the efficacy of an 8-week variant of the MBSR program in ameliorating anxiety symptom severity in 41 patients who fulfilled DSM-IV diagnostic criteria for either GAD or PD. Twenty one patients were assigned to the MBSR group while the remaining 20 were assigned to an active control group in which they were educated about the biological aspects of anxiety disorders for one hour per week. In comparison to the education group, the MBSR group displayed significant improvement in anxiety symptoms on a range of outcome measures, such as the HAM-A. Koszycki et al. [132] conducted a study which directly compared the efficacy of an 8-week course in MBSR with 12-weeks of group CBT in 53 patients with chronic SP. Participants in both treatment groups were found to improve significantly over the course of treatments, however, both patient and clinician-rated measures of social anxiety were found to be significantly lower in the CBT group in comparison to the MBSR group at endpoint. The response and remission rates were also found to be significantly higher for the CBT group in comparison to the MBSR group, although the reported response rate of 45% for the completer sample in the MBSR group is encouraging [132]. In a more recent study Vøllestad et al. [133] investigated the efficacy of 8-week MBSR in reducing anxiety amongst 39 patients with PD, SP, or GAD, in comparison to a wait list control group of 37 participants. A significant difference between treatments in favor of MBSR was found on the primary anxiety outcome. Further, all treatment gains were found to be maintained at 6-month followup in the anxiety disorders group [133].

 It has been argued that mindfulness practice may be of some benefit in OCD [134]. However, there is a scarcity of empirical research to date to investigate the efficacy of mindfulness interventions for OCD, despite the fact that some clinicians have claimed a degree of success using mindfulness approaches with their patients [134, 135]. A small pilot study by Hanstede et al. [136] investigated the efficacy of eight meditation group meetings (similar to MBSR) with participants experiencing OCD symptoms; eight participants were assigned to the mindfulness training while nine participants were assigned to a waiting-list control group. The mindfulness intervention was found to significantly reduce OCD symptoms by enhancing the mental ability of “letting go”. However, in the absence of a formal diagnosis of OCD and a broadly recognised outcome measure it is difficult to draw firm conclusions from this study.

#### 3.4.3. Mechanisms of Action

A number of mechanisms have been proposed whereby meditation and mindfulness techniques may ameliorate anxiety symptoms. First, through meditation training physiological arousal or negative thoughts may become viewed increasingly as transient events that come and go, decreasing the propensity of these thoughts to trigger secondary reactions that increase subjective distress [137]. Second, meditation has been proposed as a form of gradual exposure therapy, whereby prolonged exposure to anxiety-provoking thoughts and images may foster habituation [138, 139]. In this regard, Rapgay et al. [140] argue that “classical mindfulness,” with a greater emphasis on concentration to acquire direct experience, may be more effective for the treatment of GAD than MBSR because it involves greater exposure and habituation to threatening stimuli, rather than discriminate analysis.

The benefits of meditation and mindfulness are also now beginning to be understood at a neurobiological level. Neuroimaging studies have provided evidence to suggest that regular meditation is associated with a number of changes to gray matter morphology using magnetic resonance imaging (MRI). A study by Lazar et al. [141] reported that regular insight meditation practice was found to be associated with increased cortical thickness in a number of brain regions associated with attention, such as the prefrontal cortex and right anterior insula. Differences in prefrontal cortical thickness were also found to be more pronounced in older participants, which the authors interpreted as evidence that meditation may offset decline in cortical thickness associated with aging [141]. A number of other meditation practices have also been investigated, with the hippocampus and the right anterior insula found to be the most consistently implicated regions for group differences [142]. Finally, there is emerging direct evidence suggesting meditation may also influence neurotransmitter release, with intriguing research by Yu et al.[143] reporting that 20 minutes of focused attention on breathing movements in the lower abdomen is associated with significantly increased levels of oxygenated haemoglobin in the anterior PFC, as measured by near-infrared spectroscopy, as well a significant increase in whole blood serotonin (5-HT) levels at 5 minutes and 30 minutes after meditation [143]. 

#### 3.4.4. Yoga and Tai Chi

Yoga and Tai chi are mind-body practices that both have a long standing history of use. Yoga is generally defined as a practice which consists of three components: gentle stretching, exercises for breath control, and meditation as a mind-body intervention [144]. Tai chi is practiced in China as both a form of exercise and as a martial art and involves moving from a standing position through a series of postures like a choreographed dance. Sequences of postures are known as “forms”, which require considerable time and concentration to master [145].

In a systematic review of yoga for anxiety, Kirkwood et al. [144] assessed eight studies for evidence of efficacy in the amelioration of anxiety. Positive results were reported for the effects of yoga treatments of durations up to three months, although many of the studies suffered from methodological limitations such as inadequate randomisation and high dropout rates. Firm conclusions were also hindered by the fact that there was a large diversity in the conditions studied, including OCD, examination anxiety, snake phobia, and the outdated diagnoses of anxiety neurosis and psychoneurosis [144]. It is interesting to note that the most rigorously conducted study was in 22 adults with OCD, as diagnosed by DSM-III-R. In this small study, three months of yoga treatment was found to provide a significantly greater reduction on the Yale-Brown Obsessive Compulsive Scale (Y-BOCS) and SCL-90-R at endpoint in comparison to the relaxation response and mindfulness meditation control group [146].

A recent systematic review of the effects of Tai chi on psychological health and well-being was conducted by Wang et al. [147]. While not relating directly to clinically diagnosed anxiety disorders per se, the authors identified two RCTs and six nonrandomised studies which reported Tai chi to be associated with a significant reduction in anxiety when practiced two to four times a week (30 to 60 minutes/time) for five to 24 weeks. The overall effect size (Hedge's *g*) associated with reduction in anxiety symptoms between groups was reported to be 0.66. However, it should be noted that the 359 participants included in the metaanalysis presented with a diverse range of health conditions. While these preliminary findings are intriguing, further research using individuals with formally diagnosed anxiety disorders is required in order to properly test for the efficacy of Tai chi in ameliorating anxiety in clinical populations. In regards to the putative mechanisms of action of yoga and Tai chi in reducing anxiety, further investigation is also required in order to properly differentiate the effects of these meditative exercise practices compared to normal physical activity or other relaxation techniques.

#### 3.4.5. Conclusions

In summary, while research into the effectiveness of meditative techniques in the treatment of anxiety is still in its infancy, a number of studies in clinical populations have provided encouraging preliminary data of a potential benefit for MBSR technique in GAD, SP and PD, and insufficient but encouraging evidence for yoga and Tai chi for the treatment of general anxiety. However, for OCD, it is difficult at this stage to properly gauge the efficacy of these techniques due to the scarcity of current research using mindfulness interventions. 

### 3.5. Diet and Nutrition

#### 3.5.1. Overview

Public health and governmental agencies support a balanced diet low in processed foods and rich in fruits, vegetables, whole grains, legumes, fish, and lean meats as part of a healthy lifestyle [148]. Such a diet reduces the risk of developing numerous medical disorders including cardiovascular disease [149] and some cancers [150]. There is now emerging evidence that diet also affects our mental health, although the relationship appears to be complex and bidirectional. Many would recognise that changes in stress levels influence our dietary choices. In a study of self reported eating behavior, Oliver and Wardle [151] noted that although people differentially increase or decrease their food intake when anxious and stressed, the choice of food appears to consistently move away from normal meal-type foods toward high fat, high palatable snacks. This finding appears independent of gender or dieting status. Such changes in stress induced dietary choices have been extensively replicated [152]. However, this phenomenon may not necessarily extend to people with chronic mental disorders, such as MDD, as some may in fact better their diet in an attempt to improve their mental health [153].

#### 3.5.2. Epidemiological Evidence

Research into the role of diet on mental health is still developing and few studies have assessed this in anxiety-disordered populations. However, literature reporting on the relationships between diet quality and anxiety in population-based observational studies does provide some insight. The first study to examine the association between whole diet and clinical anxiety and depressive illnesses was conducted in population-based sample of Australian women [153]. In this cross-sectional study, women scoring higher on a “traditional” (healthy) dietary pattern, comprising fruits, vegetables, wholegrains and lean red meats, were less likely to have either an anxiety disorder or MDD or dysthymia, assessed using a gold-standard clinical interview. In this study, higher scores on a “Western” (unhealthy) dietary pattern were associated with an increased likelihood of depressive illness (OR: 1.38), but not anxiety disorders. Similarly, in a cross-sectional analysis of a large sample of middle-aged and elderly adults from Norway, higher scores on an *a priori* diet quality score (comprising vegetables, fruits, wholegrains, fish, and nonprocessed meats) was found to be associated with reduced likelihood of anxiety, measured on the HADS, in women (OR: 0.77), and with reduced case-level depression in both men (OR: 0.83) and women, (OR: 0.71) before and after adjustment for age, income, education, physical activity, smoking, and alcohol consumption [154]. Conversely, consumption of a “Western diet” (comprised of processed meats, pizza, salty snacks, chocolates, sugars and sweets, soft drinks, margarine, French fries, beer, coffee, cake, and ice cream) was associated with significantly increased likelihood of anxiety, but not depression, in men and women [154].

#### 3.5.3. Mechanisms of Action

Although incompletely understood, numerous studies provide some insight into the mechanisms of the diet-anxiety interaction. As in humans, increased stress in rats is associated with increased consumption of highly palatable foods [155]. In the “drive induction hypothesis” [156] this association is thought to result from the effects of stress induced elevated glucocorticoids (GC). Elevated GC are hypothesised to cross the blood brain barrier and act directly on central pathways mediating appetite behaviors (increasing motivation for consumption). As this occurs, peripheral elevations of GC facilitate increased negative feedback blunting central stress responses. There is some evidence that intake of high fat diets may provide selective protection against certain anxiety states. In a study of rats fed a chronic high fat diet, certain antidepressant-sensitive anxiety behaviors (e.g., from the forced swim test), and those provoked by the light-dark box test, appeared reduced but anhedonic and social avoidance behaviors remained unchanged [157]. The short-term protective effects of highly palatable food may however lead to adverse long-term consequences. Recent evidence has demonstrated that intake of highly palatable foods is associated with increased production of reactive oxygen species (ROS) [158]. Increased ROS production and subsequent oxidative stress are postulated to contribute to the development of anxiety disorders [159]. 

 In respect to which nutrients from the diet may have an anxiolytic effect, numerous studies have identified the relationship between omega-3 and omega-6 fatty acids and depression and anxiety. Decreased omega-3 and an increased omega-6 to omega 3 ratio are associated with an increased rate of depression in some studies [160, 161]. Similarly, decreased levels of omega-3 have been associated with increased SP [162], while low levels of DHA consumption exhibited a cross sectional relationship with anxiety disorders in a large population cohort (Jacka et al., 2012, unpublished data). In one recently reported RCT, supplemental omega-3 was compared to placebo in a group of medical students on depressive and anxiety symptoms and levels of various inflammatory mediators [163]. The students who received omega-3 supplementation showed a 14% reduction in lipopolysaccharide stimulated interleukin 6 (IL-6) and 20% reduction in anxiety symptoms measured on the BAI. Interestingly, no effect was found on depressive symptoms. Other dietary elements including magnesium [164] and zinc [165] also appear related to mental health status [166], and have preclinical animal model evidence of anxiolytic and antidepressant effects [167, 168], however, empirical studies have not yet confirmed any therapy effect of supplementation for anxiety disorders.

#### 3.5.4. Conclusions

While prospective studies are currently limited, the emerging body of evidence encourages the recommendation for bettering general mental health by adopting a diet rich in lean protein, complex carbohydrates, fruit and vegetables, with adequate omega 3; and low in refined carbohydrates, saturated fats, and processed foods. At present however, no clinical trial has been conducted assessing the effect of a healthy diet versus a suitable control, to establish whether dietary modification is beneficial in treating anxiety disorders.

### 3.6. Substance Use/Misuse (Alcohol, Caffeine, Nicotine)

#### 3.6.1. Overview

There is a solid body of evidence linking lifestyle factors with depression. Modifiable factors impacting on risk, course and outcome include alcohol, smoking, recreational drugs, diet, and exercise. The same is true of anxiety, although the body of clinical and epidemiological data is larger in depression than in anxiety. There is however a well-replicated relationship between anxiety disorders and substance use. The use of all drugs of dependence (smoking, alcohol, opiates) result in an enduring withdrawal state interrupted by periodic intoxications. This is mediated by the homeostatic adaptation to the persistent presence of exogenous drugs. Substance abuse can also be seen as a maladaptive coping strategy that procrastinates having to manage the source of the dysphoria or anxiety and undermines the development of adaptive strategies [169]. Substance use may mitigate anxiety symptoms in the short-term. However, the use of tobacco and/or alcohol predisposes people to the development of anxiety over time by producing chronic withdrawal symptoms, reduced health quality, and the possible precipitation of somatic or emotional symptoms that maintain anxiety [170]. It is however, important to note that not all individuals who have anxiety abuse substances; other vulnerability factors, including personality, social support, development and attachment, and genetics, mediate this relationship. From a management perspective, anxiety and concurrent substance abuse require integrated management. While there are few trials for management of comorbid substance use and anxiety [171], CBT, for example, has been shown to be useful for substance abuse related anxiety [172]. 

#### 3.6.2. Nicotine

Smoking is more common in those with anxiety, particularly PD [173], and those with anxiety smoke for longer periods, increasing the predisposition to smoking-related complications. In the Australian Survey of Mental Health and Wellbeing [174], over a fifth of adult smokers reported 12-month anxiety disorders. There was a dose dependant relationship between smoking rates and illness severity, however, smoking rates were doubled even among those with mild anxiety. Complicating management, smokers with anxiety were less likely to attempt cessation. Smoking cessation specifically for the treatment of anxiety has not been formally examined in rigorous clinical trials, however, anxiety is noted to increase withdrawal symptoms in the context of smoking cessation [175]. People with anxiety or depression are as likely as those without these symptoms to want to stop smoking, but are less likely to make a quit attempt [176]. Smoking cessation, importantly, does not appear to increase anxiety or depression after quitting and might reduce symptoms, although data are inconsistent [177–179]. Cigarettes modulate perception of emotional and somatic states via postsynaptic nicotinic receptor agonism, and ultimately downregulate dopamine D2 receptors in the nucleus accumbens, the site of the reward system [180].

#### 3.6.3. Caffeine

Caffeine is perhaps the most commonly used psychoactive substance. It is perceived to increase attention, alertness, cognition, and mood, with some individuals with dysphoric mood being predisposed to use it more heavily, tapping into its mood-elevating potential (via activation of noradrenergic and dopaminergic pathways) [181]. As a common consequence of overuse, this psychostimulant may increase arousal and subsequent anxiety and insomnia [182]. It additionally carries the potential for mild drug dependence. Those with anxiety disorders, including PD and SP, are especially sensitive to the anxiogenic effects of caffeine. The effects of oral administration of caffeine (10 mg/kg) in 17 healthy subjects and 21 patients meeting DSM-III criteria for agoraphobia with panic attacks or panic disorder found caffeine produced significantly greater increases in subject-rated anxiety, nervousness, fear, palpitations, restlessness, and tremors compared with healthy controls [183]. Caffeine also increased plasma cortisol levels in both the anxious and healthy groups. Caffeine modulates the adenosine system, while the anxiogenic potential of caffeine is influenced in part by polymorphisms of the A2A receptor [184]. While evidence supports the avoidance of caffeine in anxiety disorders, a contrary therapeutic approach involves using caffeine to provoke panic attacks (similar to inducement from exercise), in order to train the person via a caffeine “challenge test” to manage these anxiogenic symptoms [185]. While prospective studies have not evaluated the effect of caffeine reduction/avoidance in people with anxiety disorders, pragmatically, those with anxiety symptoms who have large amounts of caffeine should be counselled to moderate their intake.

#### 3.6.4. Alcohol

Approximately half of people receiving treatment for an alcohol misuse or abuse disorder also suffer with an anxiety or depressive disorder [186]. Alcohol misuse or abuse may prompt acute withdrawal anxiety, via an increased level of stimulatory glutamate, and a desensitization of GABA pathways [187]. Furthermore, long-term alcohol use may reduce levels or critical nutrients required for neurological function, such as B vitamins [188]. A metaanalysis of RCTs that reviewed additional focused treatment of alcohol use disorder in patients with comorbid anxiety disorders (GAD, PD, SP) found a significant effect for both drug and CBT interventions for reducing anxiety, with a pooled effect size (*d*) of 0.52 [189]. This effect was also significant for addressing alcohol reduction with an effect size of 0.27. 

 As anxiety disorders are also highly comorbid with alcohol abuse and dependence [190], there appears to be a bidirectional relationship between alcohol use and anxiety, such that each disorder can maintain or exacerbate the other. This relationship appears most relevant in young people, the age of genesis of most adult patterns of illness [191]. The coaggregation of these disorders has a range of potential explanations. The “self-medication” hypothesis suggests that people use alcohol to reduce anxiety, emotional distress or dysphoric affective symptoms [192], and thus patients with affective or anxiety disorders should be screened for alcohol misuse.

#### 3.6.5. Conclusions

While the link between substance misuse and generalised anxiety or anxiety disorders has been established, further therapeutic treatment approaches are required. At present, CAM treatments such as herbal medicines or acupuncture appear to not have sufficiently supportive evidence in addressing alcohol misuse or abuse disorder [193, 194]. Regardless, it is likely these may have a role as “supportive” interventions rather than treatments per se. An erudite clinical position is to encourage withdrawal/reduction of alcohol, nicotine and caffeine with appropriate health professional supervision. 

## 4. Discussion 

Across the breadth of the literature reviewed, many nonconventional interventions provide promise for assisting in reducing anxiety and treating anxiety disorders. Of the nutraceuticals reviewed, the only intervention currently supported by evidence is Kava, while preliminary evidence is extended for a range of nutraceuticals. Currently, of the CAM modalities reviewed, naturopathic medicine has one supportive un-replicated study, while acupuncture and massage have preliminary evidence, although the area suffers from poor methodology and the common use of nonclinical samples. Present evidence does not support homeopathy as a treatment to reduce anxiety. This review suggests that supportive evidence exists for the recommendation of lifestyle modifications including moderate graded exercise, mindfulness meditation techniques, and caffeine minimisation. While no prospective studies support the use of dietary modification as an intervention, the epidemiological evidence supports dietary improvement. Restriction of lifestyle vices such as alcohol and nicotine appears to be beneficial in reducing anxiety, although more data are needed, specifically the study of assisted substance-withdrawal programs on anxiety disorders, and controlled prospective studies. One consideration regarding the impact of lifestyle modification in addressing anxiety disorders that is challenging to tease apart scientifically, is the effect that by altering behavior (e.g., a new dietary pattern or exercise), this will usually cause a range of psychosocial changes that may also be therapeutic. 

 Currently, a notable gap in the literature concerns the deficit in our understanding of the impact of healthy dietary change on anxiety disorders. Surprisingly, to date there have been no quality RCTs investigating the impact of whole dietary improvement in those with an anxiety or depressive disorders. As such, further such investigations would assist in making specific dietary recommendations in mental health treatment. Regarding MBSR as a treatment, future research would benefit from the following guidelines; (i) the effects of MBSR needs to be clearly differentiated according to the varied anxiety disorders, (ii) greater effort needs to be made to include a suitable control group, and (iii) there needs to be stricter adherence to the standardised MBSR protocol together with more detailed documentation of treatment compliance. It would be valuable to also consider the efficacy of longer term meditation and mindfulness techniques in the treatment of anxiety and the prevention of relapse. Given that these techniques have traditionally been used as a daily practice over the course of the lifetime, it would be informative to conduct studies of longer duration. 

 Advice for clinicians wishing to institute lifestyle, self-help or CAM interventions in patients with anxiety disorders centers initially upon considerations such as their patients' diagnosis and state of distress, current medication, psychosocial factors, personal preferences, and financial resources. Specific considerations include assessment of a patient's physical health if prescribing exercise, as is referral for appropriate assessment. Dietary modifications should be delicately instituted as patients may find change difficult if using food as a psychological crutch. Referral to dieticians, often accessible through the public system, may be of benefit to some. Considerations regarding nutraceuticals include potential interactions with medications, thus pharmacokinetic and pharmacodynamic elements need to be assessed. Cost of these supplements is also a factor, with some high-quality standardised nutraceuticals being expensive. CAM research needs to be increased, as currently many nutraceuticals and modalities have either unreplicated studies, or studies using poor methodology. Reduction of vices such as alcohol, caffeine and nicotine may initially exacerbate anxiety and should therefore be undertaken in a graded and supported fashion. 

 Limitations to this metareview include a nonsystematic approach to data inclusion. Regardless, a narrative inclusive methodology was employed specifically to cover both an over-arching cross section of the literature, and to provide a clinically relevant narrative for readers. Only English language studies were included, which may have discounted other important evidence. A final limitation is that, as the area covered was very broad, an intricate written analysis of the data was not possible due to publication space restrictions.

 In summary, a range of CAM, lifestyle and self-help interventions hold promise as adjunctive approaches to psychological and pharmacological interventions to reduce anxiety and treat anxiety disorders. Future focus can involve the use of integrated approaches combining evidence-based approaches to address lifestyle, psychological, biological, and sociological determinants of these disorders.

## Figures and Tables

**Table 1:** Domains and individual interventions reviewed.

Lifestyle	Herbal medicine	Nutrient	Mind-body	CAM modality
Physical activity	Kava	Multi-vitamins	Meditation	Naturopathic medicine
Exercise	Passion flower	Omega-3	Mindfulness	Massage
Diet	Scullcap	Magnesium	Yoga	Acupuncture
Caffeine	Bacopa	Inositol	Tai chi	Homeopathy
Nicotine	Ginkgo			Aromatherapy
Alcohol	Lemon balm			
	St John's wort			

**Table 2:** Summary of nutraceuticals reviewed.

Nutraceutical	Study references^∗^	Study type	Evidence level	Outcomes	Duration	Sample	Mechanisms
Herbal medicine							
Bacopa (*Bacopa monnieri*)	Stough [39]	2	Limited Positive	STAI	3 months	Healthy	5HT_2c_, metal chelation, antioxidant and anti-inflammatory effects. Possible cholinergic effects
Chamomile (*Matricaria recutita*)	Amsterdam [40]	2	Limited Positive	HAM-A	8 weeks	GAD	GABA receptor binding and modification of monoamine pathways
Ginkgo (*Ginkgo biloba*)	Woelk [41]	2	Limited Positive	HAM-A	4 weeks	GAD or ADWAM	Antioxidant and anti-inflammatory properties, effects on GABA and monoamine systems
Kava (*Piper methysticum*)	Pittler [42]; Witte [43]	1	Positive	HAM-A	Various	Various	Blocks calcium and sodium channels, bind to GABA receptors, and inhibits noradrenalin uptake
Lemon balm (*Melissa officinalis*)	Kennedy [44]; Kennedy [45]	2	Limited Positive	Self-rated calmness	Acute	Healthy	Inhibition of GABA-T and MAO- A. Cholinergic receptor binding demonstrated in vitro
Passion flower (*Passiflora incanata ) *	Movafegh [46]; Akhondzadeh [47]	2	Limited Positive	Numerically rated anxiety, HAM-A	Acute, 4 weeks	Surgery patients, GAD	Binds to GABA-A receptors and modulates GABA-A and GABA- C pathways
Scullcap (*Scutellaria lateriflora ) *	Wolfson [48]	2	Limited Positive	Self-designed scale	Acute	Healthy	Possible bindings to the benzodiazepine site of the GABA-A receptor as well as antioxidant activity
St John's wort (*Hypericum perforatum*)	Kobak [49], Kobak [50]; Volz [51];	2	NonSignificant in all studies	HAM-A, LSAS, YBOCS	6 weeks	Depression, somatoform disorders, OCD, social anxiety	Modulation of monoamine, serotonin, dopamine, and norepinephrine pathways

Nutrients							
Combined nutrients	Carroll [52]; De Souza [53]; Gosney [54]; Kennedy [55]; Kennedy [56]; Smith [57]	2	Mixed Evidence	State anxiety, self-rated calmness, HADS	28 days–12 months	Healthy, healthy with premenstrual symptoms, participants in aged care	Antioxidant effects and enhanced metabolism of various neurotransmitters
Inositol	Benjamin [58]; Fux [59]; Palatnik [60]	2	Limited Mainly Positive	Severity and frequency of panic attacks, agoraphobia and OCD	4–6 weeks	Panic disorder with or without agoraphobia, OCD	Precursor in the phosphatidylinositol cycle modulating 5HT_2c_ receptors. Administration affects serotonergic, noradrenergic, and muscarinic functioning in vitro
Aromatherapy	Lee [61]	1	Limited Positive	Various. State anxiety the most common	Various	Various	Possible effect on the GABA system

^∗^First author, 1: metaanalysis or systematic review, 2: double-blind randomised controlled trial, STAI: state trait anxiety inventory, GAD: generalized anxiety disorder, GABA: gamma-aminobutyric acid, HADS: hospital anxiety and depression scale, HAM-A: hamilton anxiety rating scale, LSA: liebowitz social anxiety scale, YBOCS: yale-brown OCD scale, OCD: obsessive compulsive disorder, ADWAM: adjustment disorder with anxious mood, MAO-A: monoamine oxidase A.
